# Novel transfer learning schemes based on Siamese networks and synthetic data

**DOI:** 10.1007/s00521-022-08115-2

**Published:** 2022-12-16

**Authors:** Philip Kenneweg, Dominik Stallmann, Barbara Hammer

**Affiliations:** Machine Learning Group, Bielefeld University, Bielefeld, Germany

**Keywords:** Transfer learning, Twin-VAE, Siamese networks, Single-cell cultivation, Few-shot learning

## Abstract

Transfer learning schemes based on deep networks which have been trained on huge image corpora offer state-of-the-art technologies in computer vision. Here, supervised and semi-supervised approaches constitute efficient technologies which work well with comparably small data sets. Yet, such applications are currently restricted to application domains where suitable deep network models are readily available. In this contribution, we address an important application area in the domain of biotechnology, the automatic analysis of CHO-K1 suspension growth in microfluidic single-cell cultivation, where data characteristics are very dissimilar to existing domains and trained deep networks cannot easily be adapted by classical transfer learning. We propose a novel transfer learning scheme which expands a recently introduced Twin-VAE architecture, which is trained on realistic and synthetic data, and we modify its specialized training procedure to the transfer learning domain. In the specific domain, often only few to no labels exist and annotations are costly. We investigate a novel transfer learning strategy, which incorporates a simultaneous retraining on natural and synthetic data using an invariant shared representation as well as suitable target variables, while it learns to handle unseen data from a different microscopy technology. We show the superiority of the variation of our Twin-VAE architecture over the state-of-the-art transfer learning methodology in image processing as well as classical image processing technologies, which persists, even with strongly shortened training times and leads to satisfactory results in this domain. The source code is available at https://github.com/dstallmann/transfer_learning_twinvae, works cross-platform, is open-source and free (MIT licensed) software. We make the data sets available at https://pub.uni-bielefeld.de/record/2960030.

## Introduction

Systematic single-cell studies of live cell imaging from microfluidic single-cell cultivation (MSCC) works with high spatial and temporal resolution of cellular behavior. So far, analysis of images like these has mostly been performed manually or is assisted by technological aiding systems, yet requiring human experts and therefore extensive human labor to create annotations of images; clearly, this procedure is not feasible in many cases, and it creates the need for different, more affordable and automated computer vision solutions [24].

The current state of the art for computer vision tasks and image processing that does not require human labor are convolutional deep neural networks [8]. These are also used extensively in the biomedical domain [18]. Especially, approaches to track cells in images [15] have been an ongoing field of study in recent years. However, optimisation for this task has proven to be a very cumbersome challenge which remains prone to errors.

The proposed benchmark suite [26] allows comparing different imaging technologies and extrapolation of the strengths and limitations of diverse methods for cell tracking, none of which are deemed as final solution for this task, even those with added interaction by bioimage analysis experts [1] or distributed work of manual labeling [7].

In this contribution, we address a challenging task in biomedical image analysis by means of specific and adapted transfer learning technologies. Related work in this field includes Brent et al. [2] which used transfer learning to predict microscope images between different imaging technologies, however without sufficient incorporation of the vast diversity of cell imagery and characteristics. The approach by Falk et al. [5] provides one of the few toolboxes for cell tracking, albeit adherent, rather than suspension cells. It allows transfer learning based on given models and novel data, whereby data set enrichment technologies limit the number of required samples.

In contrast to already reported single-cell cultivation studies [4] and [12], where adherent growing cell lines are the focus of investigation, we address the scenario of more complex suspension cells, with their circular basic shape but ever-changing contour due to vesicle secretion and additional challenges like cell movement and floating within the experiment chamber, which renders analysis tools of adherent cells deficient. These cells growing in suspension comes with different and challenging obstacles to achieve automation of analysis, which will be described in Sect. 2.

In this work, we want to make use of a network trained on one microscopic image type and adapt it to provide sufficiently accurate cell counting for a different microscopy technology, where no trained network exists due to the lack of annotated data. We particularly focus on mitigating human labor for annotations. Our previously introduced deep twin auto-encoder architecture Twin-VAE [22] is trained on data stemming from one imaging modality and thereafter transferred to the similar yet different domain of the other microscopy technology. This training procedure greatly reduces the need for natural, labeled data, by using synthetic, auxiliary training data, for which the ground truth is known and which is easy to obtain in this setting, since the Twin-VAE does not require the images to be rendered realistically in every regard, such as morphological details.

In the following, we will first describe the specific application domain from biotechnology, the underlying machine learning challenge, and the deep Siamese network architecture which will be used for transfer learning. Afterwards, we elaborate the details of the proposed transfer learning scheme, as well as perform an analysis of how the unique architecture used affects the transfer learning procedure. Its performance is evaluated for real-data sets and using ablation studies, as well as comparison to state-of-the-art alternatives and baselines. A discussion concludes the contribution.

The application area in question is a prime example of a domain, where the state-of-the-art Image processing techniques do not work sufficiently well due to very little texture and other visual characteristics of the images, described in Sect. 2. In addition, there exists no Deep Learning Models which easily and efficiently solve the task, as shown in [22] by comparing to EfficientNet [23], and Watershed methods [17] and shown here later by comparing to BigTransfer [11] and our previous work. The emergence of more data in such specialized domains like this makes it important to provide an easy-to-use system which has a high performance and enables automation of processes involving this kind of data.

Thus, the contribution and novelty of our work is as follows:

- We improved performance and lowered computational complexity (outperforming the original work [22])
- We build an efficient transfer pipeline and showed on two microscopy datasets empirically that it outperformed a variety of methods, including state-of-the-art image processing.
- By performing extensive ablations studies we gained insight into which parts of the architecture contains representations which are beneficial for the transfer learning ability of the network. Thus, we are contributing to the debate how deep neural networks represent information. [9]

## Materials and methods

### MSCC and live cell imaging data

**Fig. 1:**
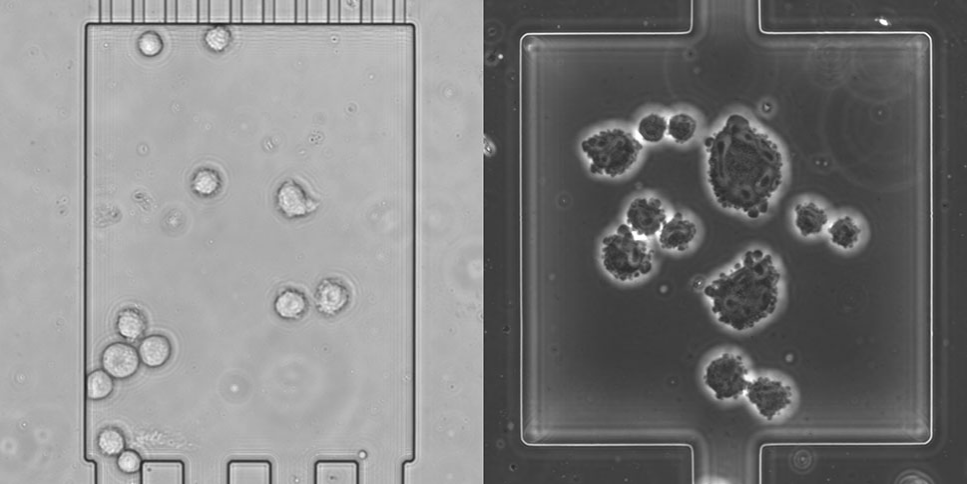
Samples of data from the two microscopy technologies. Bright-field microscopy (left), phase-contrast microscopy (right). The data has been preprocessed in the form of chip drift removal and orientational stabilization (translational and rotational) and a crop to square the images that allows further cropping by data augmentation techniques. The cell counting module has to differentiate between the cells, smudges, chamber and background

The image data which is used in this study was obtained by single-cell cultivation of mammalian suspension cells as shown before [20]. CHO-K1 cells were cultivated in polydimethylsiloxane (PDMS)-glass-chips and constantly provided with nutrients by perfusion of the microfluidic device. The goal of an automated analysis of such data is an automated extraction of important parameters of the observed dynamics, such as cell growth. Since many important parameters can be estimated based on the number of cells at a specific time point, the number of cells constitutes a key quantity and are taken as target labels. The data used in this work consists of multiple parts, characterized (1) by the according microscopy technologies, bright-field microscopy and phase-contrast microscopy, (2) by the type of data, natural or synthetic, i.e. the original data or data which are generated and added to the original one within the learning pipeline, as described later, (3) by existence of a label and (4) the usage of that data for training or testing. Example images of both modalities are shown in Fig. 1.

Table 1 shows an overview statistic of all data sets. The Nat set contains the aforementioned natural images, Syn the synthetic ones. The BF tag declares bright-field microscopy images, consisting of 12 experiment scenarios with 956 overall images, of which a label (i.e. cell count) exists for 7.5% of the training images. The PC tag is denoting phase-contrast microscopy images which consist of 37 experiment scenarios, accumulating to 3976 used images with a labeling rate of 6.2% for the training data. The labels were created by hand in a nearly regular interval over the experiment scenarios for all natural data sets, however images were removed beforehand, if they had more than 30 cells, since the expected outcome of the cultivation experiment is already determined at this point.

**Fig. 2:**
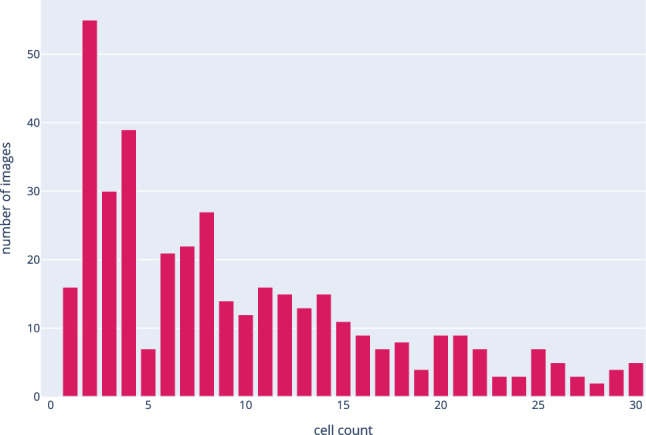
Visualization of distribution of images by cell count for the merged data sets Nat-PC-L-Te and Nat-PC-U-Te. We discard data with higher cells counts than 30, because they are irrelevant for the cultivation experiments

In the upcoming analysis, we focus on the transfer from the larger data set Nat-PC to the smaller set Nat-BF, since this is the common way to apply transfer learning. The phase-contrast imagery also contains more variation of the biological processes, which makes phase-contrast microscopy arguably more popular than bright-field microscopy. Our experiments also contains transfers from bright-field microscopy to phase-contrast microscopy to show the robustness of the technique. Figure 2 shows the distribution of images against the cell counts in them for Nat-PC. A clear trend towards images with low cell counts can be seen.

This can be taken into account for optimization of the transfer learning methodology, since it can be assumed that data of this type has a similar distribution, particularly in the light of exponential growth rates and the presence of failing cultivations. Most of the labeled images from (L-Te) are used for testing the cell count prediction and the reconstruction, rather than used during training (L-Tr). This is done, because we focus on a method that reliably works on small amounts of labeled data. Further unlabeled test data (U-Te) is used to evaluate the reconstruction only, since (L-Te) remains too small to include a broad overview of the different chamber situations (clumping, overlapping, escaping etc.) to be confident about the stability of the performance for a convolutional network.

**Table 1:** Overview of data sets used. The Nat tag indicates natural data, Syn represents synthetic data. BF classifies the bright-field images, PC the phase-contrast ones. L and U marks labeled and unlabeled data and lastly Tr and Te separate the data into training and test data

Abbreviation	Type	Technique	Label	Usage	Size
Nat-BF-L-Tr	Natural	Bright-field	Yes	Training	281
Nat-BF-U-Tr	Natural	Bright-field	No	Training	2188
Nat-BF-L-Te	Natural	Bright-field	Yes	Testing	290
Nat-BF-U-Te	Natural	Bright-field	No	Testing	224
Nat-PC-L-Tr	Natural	PBhase-contrast	Yes	Training	209
Nat-PC-U-Tr	Natural	Phase-contrast	No	Training	2943
Nat-PC-L-Te	Natural	Phase-contrast	Yes	Testing	398
Nat-PC-U-Te	Natural	Phase-contrast	No	Testing	394
Syn-BF-L-Tr	Synthetic	Bright-field	Yes	Training	2469
Syn-BF-L-Te	Synthetic	Bright-field	Yes	Testing	514
Syn-PC-L-Tr	Synthetic	Phase-contrast	Yes	Training	3152
Syn-PC-L-Te	Synthetic	Phase-contrast	Yes	Testing	792

### Synthetic data

As our task is reliable cell counting for suspension cell microscopic images and given data is often limited and with only few manual annotations, retraining a deep neural network for every new set of data is inadequate and delivers deficient accuracies for the task. To overcome this limitation, we transfer a trained model which achieves high accuracies on its original task to the newly presented task.

Since the learning methodology is semi-supervised, our formerly introduced Twin-VAE [22] will be used as a basis to propose a novel transfer learning method to mitigate the aforementioned complications. Here, synthetic data are used as an auxiliary training set Syn which is also used for transfer learning. We evolve on the Siamese architecture, which inherently solves the task of abstraction from the synthetic nature of data set enrichtments.

**Fig. 3:**
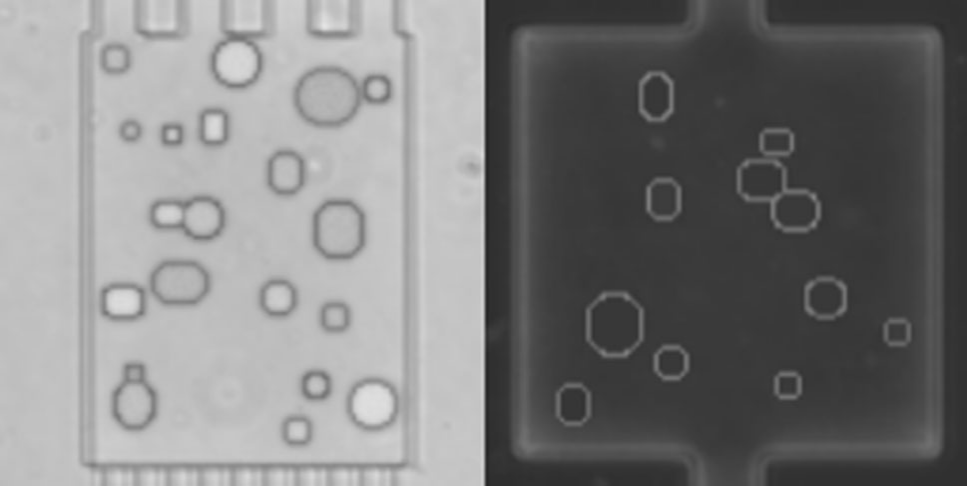
Random samples of synthetic data from the sets Syn-BF-L-Tr and Syn-PC-L-Tr. Sample of bright-field microscopy on the left and of phase-contrast microscopy on the right. The images are dimensionless and therefore do not show scale bars. The theoretical cell sizes are identical to natural data cells, but is of no importance for the work

The synthetically generated data are visually simplified (constant background, ellipsoidal cells) to allow the loss construction to focus on the regression task rather than the intricate reconstruction of arbitrary visual cell membranes and organelles. This is done by drawing cells as ellipsoids, varying some attributes like their brightness, size and blurriness of edges. For further detail, see the original Paper [22]. Reconstructions of real appearances from synthetic data, while interesting to suggesting inherent stability, are not of importance for a high accuracy on the task. Ground truth labels are known for synthetic data, because it is based on pre-defined geometric style modeling, neglecting texture and complex morphology. Thereby, geometric heterogeneity of this data is simplified compared to real data, examples of which can be seen in Fig. 3.

Synthetic data allows for creation of a large variety of independent image samples that are correlated but not identical to the appearance of natural data. Unlike popular data set enrichment technologies, the amount of data can freely be determined since it is independent of the amount of real data, and representatives of any type of underlying label can easily be generated. We show that our Twin-VAE architecture is successfully trained and improved in accuracy like this in Sect. 2.3.

Table 1 lists the synthetic data as Syn, concatenated by the microscopy technology category BF or PC accordingly. The U or L declaration tells if the data is labeled and the table separates them between training (Tr) and test (Te) images. The cell distribution in these images was chosen to be close to that of the Nat data sets for the sake of resemblance and because of the criticality of correct cell counts on low cell count imagery. Synthetic images are generated with seed consistency, i.e. in such a manner that ensures reproducibility and can be generated in arbitrary amounts, however larger amounts of synthetic data will increase training time nearly linearly, while improving performance with diminishing returns as will be shown in Sect. 5. For the sake of computational time, the default Syn set sizes roughly match the corresponding number of natural images. However, sets with different amounts have also been created for accuracy comparisons (Fig. 12). The background is created by the mean value of the entire natural training data set showing less than 5 cells to assure high visibility of empty background and to remove the smudges that appear in only a few experiment scenarios. The working resolution, the synthetic data are generated in is 128 by 128 pixels, matching the resolution of the architecture’s input size described in Sect. 2.3 and their file size is about 8 MB per 1000 images.

The virtual generator is highly adjustable, creating images with a given distribution of cell counts, overlapping of cells, variations of the brightness of the cell’s inner organelles, their membrane silhouette and the background. More complex visual fidelity of natural data such as ongoing cell divisions can also be mirrored by a combination of these mechanics, e.g. by creating a small overlap together with more noisy cell borders. Smudges, as in Fig. 1, have not been inserted, since they are an interference factor and likely only hinder the training process. The cells’ shape has been simplified to deformed ellipsis to roughly match the shape of the natural cells. Noise, individual luminance per cell, and multiplication with Gaussian filters of random strengths have been added to increase the variety of cells in the data. We ensure easy adjustability of the generation mechanism to natural cells in other data sets, that have different shape characteristics.

### Network architecture and training

#### Siamese architecture

We use a novel deep Siamese twin architecture that separates the input data for training depending on its origin, thus circumventing the problem of differences in appearance of synthetic and natural images. This approach requires that the architecture creates a tightly coupled shared inner representation of the different data sources to achieve low training losses and good generalization ability for semi-supervised setups.

**Fig. 4:**
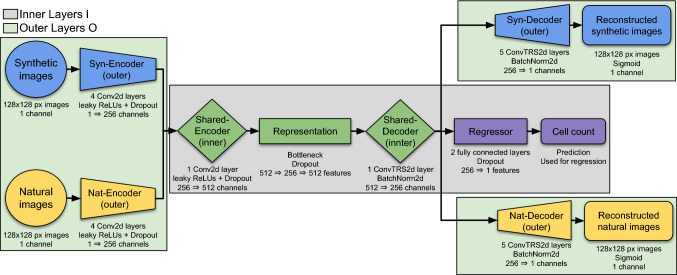
Visualization of the Twin-VAE architecture. The blue elements handle synthetic data, while the yellow elements handle natural data. The green elements are shared between the two VAEs and contain the inner representation of the cell imagery, while the purple elements result in an estimation of the cell count

For this, two identical variational autoencoders (VAE) are created for the two data sets. They share the weights of their last encoding layer, the first decoding layer and the small hidden layer in-between (see Fig. 4). VAEs constitute a state-of-the-art solution for generalized few-shot learning [21] and weight-sharing has been used to reduce neural network sizes and to improve test performance beforehand [25].

Specifically, in our setup, one of the VAEs works on synthetic data only (*VAE-syn*), while the other one uses natural data only (*VAE-nat*). The non-shared outer layers account for the different visual characteristics of synthetic and natural data, while the shared inner layers are enforced to create a common representation of relevant image characteristics. By adding a two-layer deep fully connected neural network regression model for the cell counting task, the architecture works in a supervised manner for data for which the label are known, based on the shared representation of the VAEs. Cell detection by regression has been shown to work well for other (less demanding) tasks [27, 28]. Our architecture therefore addresses two objectives simultaneously:

1. Mostly unsupervised encoding and decoding of natural and synthetic input images using a shared representation.
2. Supervised counting of the cells for both natural and synthetic images.

#### Loss

Given an input image $x$ of pixels, a label (i.e. cell count) $l$ between 1 and 30 and a type $t\in \{\mathfrak {n}, \mathfrak {s}\}$, representing the fact whether the image is natural or synthetic, we obtain a reconstruction loss ${{\,\textrm{Rec}\,}}(x)$ of the VAE, a regression loss ${{\,\textrm{Reg}\,}}(x, l)$ of the task at hand, such as cell counting, and a distributional regularization loss ${{\,\mathrm{\mathcal {D}_{KL}}\,}}$, which aims for a homogeneous representation of synthetic and real data in the embedding space of the VAE. We combine these losses to form our twin loss ${{\,\mathrm{Twin_{loss}}\,}}(x, l, t)$ with weighting factors $C_{{{\,{\rm Rec}\,}}}^{t}$, $C_{{{\,\rm{Reg}\,}}}^{t, l}$, and $C_{{{\,{\mathcal {D}_{KL}}\,}}}^{t}$, respectively, which allows us to balance image reconstruction fidelity ($C_{{{\,\rm{Rec}\,}}}^{t}$), regression performance ($C_{{{\,\rm{Reg}\,}}}^{t, l}$) and distributional stability ($C_{{{\,\mathrm{\mathcal {D}_{KL}}\,}}}^{t}$) and therefore to maximize the impact of regression errors on the loss. Furthermore, it allows us to gracefully handle input images without known cell counts by setting $C_{{{\,\rm{Reg}\,}}}^{t, l}$ to zero:

1 $$\begin{aligned} \begin{aligned}&{{\,\mathrm{Twin_{loss}}\,}}(x, l, t) \\&\quad =C_{{{\,\rm{Rec}\,}}}^{t} \cdot {{\,\textrm{Rec}\,}}(x) + C_{{{\,\rm{Reg}\,}}}^{t, l} \cdot {{\,\textrm{Reg}\,}}(x, l) + C_{{{\,\mathrm{\mathcal {D}_{KL}}\,}}}^{t} \cdot {{\,\mathrm{\mathcal {D}_{KL}}\,}}(x) \end{aligned} \end{aligned}$$

During our experiments, the mean-squared error (MSE) $||x-d(x)||^2$, where $d(x)$ is the reconstruction of the input image $x$ and $||l-r(x)||^2$, where $r(x)$ is the estimated cell count, yielded the best results respectively, when used as ${{\,\textrm{Rec}\,}}(x)$ and ${{\,\textrm{Reg}\,}}(x, l)$ for training on phase-contrast data, and as ${{\,\textrm{Reg}\,}}(x, l)$ for bright-field data. However, for bright-field data the binary cross entropy (BCE) $-l\cdot \text {log}(r(x))+(1-l)\cdot \text {log}(1-r(x))$ turned out to be the superior choice for ${{\,\textrm{Rec}\,}}(x)$ and was often resulting in just slightly worse results than the MSE for phase-contrast data. The ${{\,\mathrm{\mathcal {D}_{KL}}\,}}$ is applied as the Kullback–Leibler divergence (KLD) of the standard VAE model ([10]) and is obligatory to enforce generation of latent vectors with sufficient similarity to a normal distribution. The weighting factors $C_{{{\,\rm{Rec}\,}}}^{t}$, $C_{{{\,\rm{Reg}\,}}}^{t, l}$, and $C_{{{\,\mathrm{\mathcal {D}_{KL}}\,}}}^{t}$ are carefully chosen for training, punishing incorrect cell count predictions especially on natural data, while relaxing the importance of visual reconstruction. Details on this are provided in the following section.

#### Neural network structure

The outer, non-shared part of the encoder is composed of four two-dimensional convolutional layers with kernel size 5 and a stride of 2, initialized with an orthogonal basis [19]. Inbetween the layers, leaky rectified linear units (ReLU) with a leakiness of 0.2 are activated, together with a dropout of 0.1. Channel amounts used for the convolutions are in order: 32, 64, 128 and 256 for the encoders. The inner, shared part of the encoder consists of an additional two-dimensional convolutional layer with identical properties and 512 channels. The layer is followed by the bottleneck, consisting of three fully connected layers of sizes 512, 256 and 512 again, each with a dropout of 0.1. The inner, shared part of the decoder has 256 channels, contains a two-dimensional transposed convolutional operator layer with identical kernel size and stride as in the encoder, and is followed by a batch normalization over a four-dimensional input and a leaky ReLU with the same leakiness. The outer, non-shared part of the decoder consists of five layers of kernel sizes 5, 5, 5, 2, 6, following the convention of a small penultimate followed by a bigger last layer, keeping the stride of 2 except for the fourth layer using a stride of 1, the same leaky ReLUs and a sigmoidal activation function at the end. Additionally, a branch of fully connected neurons for the regressor consisting of two layers of sizes 256 and 128 is being fed by the output of the shared part of the decoder, uses linear layers and a constant dropout of 0.1.

The architecture is using the Adam optimizer for phase-contrast microscopy data, and the rectified Adam (RAdam) [13] optimizer for bright-field data. The combination of the decoder loss factor $C_{{{\,\rm{Rec}\,}}} = 100$, the regressor loss factor $C_{{{\,\rm{Reg}\,}}}=3$ and the KLD factor $C_{{{\,\mathrm{\mathcal {D}_{KL}}\,}}}=2$ yields the best results for phase-contrast data. For the BCE, the decoder loss factor is not constant, but decays over time with a rate of $3\times 10^{-5}$ per epoch, since the BCE does not decrease significantly within the training process, but needs to decrease over time to amplify the importance of low regression losses ${{\,\textrm{Reg}\,}}(x, l)$.

While it seems counter-intuitive that $C_{{{\,\rm{Rec}\,}}}$ is bigger than $C_{{{\,\rm{Reg}\,}}}$ and $C_{{{\,\mathrm{\mathcal {D}_{KL}}\,}}}$, it is caused by the MSE for pixel data getting very small on normalized images. KLD is supposed to stay relatively small. While it is required to enhance the quality of the distributions, it should not impact the training of cell predictions and image reconstructions too much by unfortunate sampling from the latent vector, however it has to be impactful enough to enforce natural and synthetic data into similar representations in the inner layers.

Since training is done over thousands of epochs, a soft weight decay of $1\times 10^{-5}$ per epoch is added, combined with a fixed learning rate of $1.3\times 10^{-4}$. A delayed start for the regressor is used to allow for pure image reconstructions to contain meaningful images, ensuring the representation of information of existent cells in the representation before the regressor has to extract that information. A delay of 100 epochs has been used to achieve the results presented in Sect. 5.

A batch size of 128 for the phase-contrast images and 64 for the bright-field images works best, and the training runs for up to 50.000 epochs, unless early stopping conditions abort it.

#### Data augmentation

To maximize the use of the limited amounts of natural data, multiple data augmentation techniques are combined and applied to the data. Randomly occurring horizontal and vertical flips, possibly combined with a random crop of the image of scale 0.9 combined with a resize to its original, meaning the images get randomly cropped to 115 pixels in width and height, and then scaled back to 128 pixels.

The crop adds difficulty to the cell detection process by partially cropping cells out, however it proved helpful as long as the crop is not too strict and cuts away cells completely. Then, a 90 degree rotation is applied at random and a zero-centered noise map is generated and added to the image with a small amplitude factor. Additionally, small rotations of 0 to 5 degrees are added before the crop, to spread cell occurrence even more. The crop will then mostly remove the undefined parts of the image, that are created when rotating non-circular images.

### Image reconstruction

Although our goal is automatic counting of cells, our loss from Eq. (1) includes a term for image reconstruction. The reasoning behind this decision is that analysis of the reconstruction abilities of Twin-VAE is only possible with this loss. Furthermore, the loss enables us to check if the learned shared representation is meaningful by checking the correlation between the visual existence of cells in the image reconstructions and the actual cell count.

During training of Twin-VAE, the natural input images are first processed by a specialized encoder, followed by a shared encoder and decoder of the two twins, and finally reconstructed by a specialized decoder (see Fig. 4). Synthetic data is handled equivalently. The learned inner representation must be shared and similar between the two types of data for (1) the regression to work as intended and (2) the cell counting in natural images to benefit from synthetic data as much as possible. Verification of this is done by encoding a natural image with the appropriate encoder but performing the reconstruction with the decoder that is intended and trained for synthetic images and vice versa. In the following, we demonstrate exactly this.

**Fig. 5:**
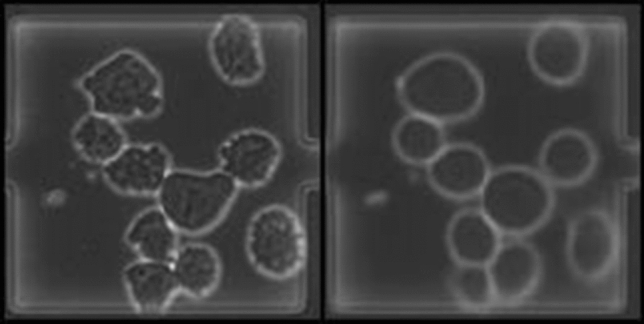
Example of a perfect synthetic-looking reconstruction (right) of a natural image (left) from Nat-PC-L-Te. The cell counts match exactly and the position as well as size of cells are preserved. While the smudge on the left is recreated visually, it does not lead the regressional part of the Twin-VAE to a wrong cell count

**Fig. 6:**
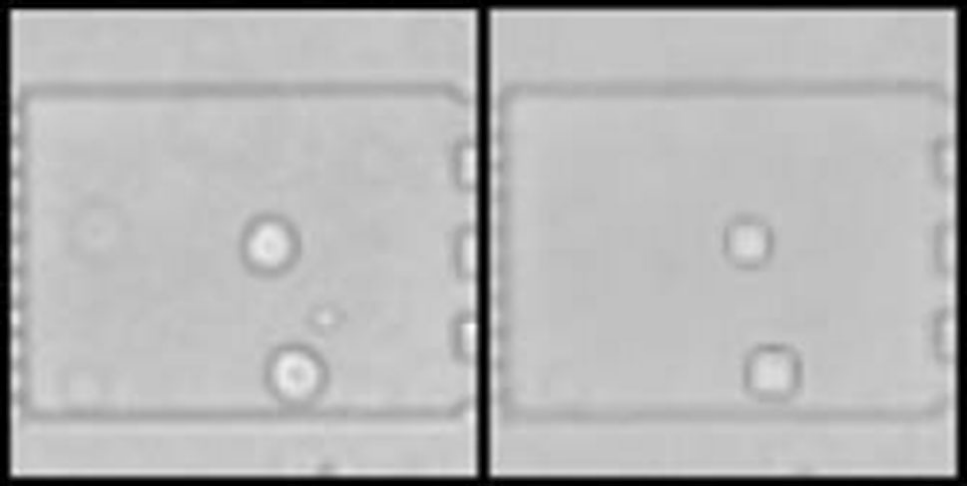
Example of a perfect synthetic-looking reconstruction (right) of a natural image (left) from Nat-BF-L-Te. The cell counts match exactly and the position as well as size of cells are preserved. For this data set, where smudges are more faint, they don’t get reconstructed usually

In Figs. 5 and 6 we show examples of perfect translations, where a natural image is encoded and subsequently decoded as a synthetic image. The cell count is unchanged, the cell prediction matches the actual existence of cells and the position and size of cells are also retained, while the overall appearance is simplified, however Twin-VAE has learned to remove noise and condense the information down to what is required and helpful to count cells.

**Fig. 7:**
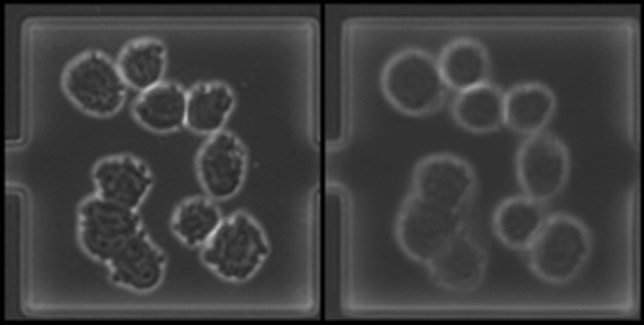
Example of a faulty synthetic reconstruction (right) of a natural image (left) from Nat-PC-L-Te. The human expert determined the cell count to be 10, the prediction differs by one. The reconstruction shows a merge of the top two cells in the bottom-left triple of cells. The two cells clump together in such a way, that there is almost no visual indication of a border between them, especially missing the usual bright boundary around cells that can be seen around the rest of the cells

**Fig. 8:**
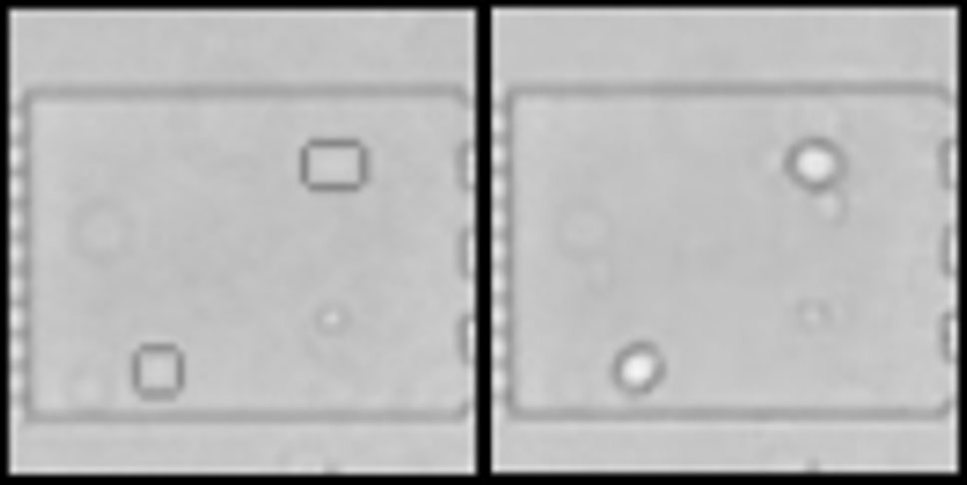
Example of an accurate, natural-looking reconstruction (right) of a synthetic image (left) from Syn-BF-L-Tr. Cell counts match exactly, position and size of the cells are preserved. While these conversions are not mandatory for the transfer process, they ensure representational consistency on a visually comprehensible level

Even when Twin-VAE does not translate an image perfectly, the reconstruction can be useful to understand where an error occurs. In Fig. 7 we show an example where two cells that are very close together are interpreted and reconstructed as a single cell. As well as translating images from natural to synthetic-looking, Twin-VAE can perform the inverse translation from synthetic to natural-looking as well. We provide an example in Fig. 8.

### Baselines

We implement two different methods to serve as baselines for our evaluation.

The first is Twin-VAE which already outperforms classical baselines like *EfficientNet* [23] and Watershed [17]. We compare our new Transfer Twin-VAE to Twin-VAE and BigTransfer, commonly shortened to BiT [11]. Twin-VAE is a previous work of ours upon which Transfer Twin-VAE and the variation double Transfer Twin-VAE build. It consists of the same architecture but is trained upon a single dataset consisting of natural and synthetic images. Furthermore, less extensive hyperparameter tuning was performed on Twin-VAE due to longer training times.

The second method we compare to is a transfer learning pipeline from Kolsenikov et al. called BiT that produces state-of-the-art classification results on Cifar-100 and similar datasets in the few shot case (1–10 examples per class). BiT consists of the classical ResNet [6] architecture but with very long pre-training times on large image corporas and a custom hyperrule that determines the training time and learning rate during transfer dependent on the new dataset size. Since the data augmentation applied during BiT is not immanently applicable in the cell counting case, we used the same data augmentation as in our own method. We tested changes to the hyperrule presented in BiT but did not find any significant improvements, therefore used the values provided by the authors.

## Transfer learning methodology

In this chapter, we will describe various experiments to determine which transfer methodology is suited best to the Twin-VAE architecture and the final transfer learning methodology used.

### Transfer method

**Fig. 9:**
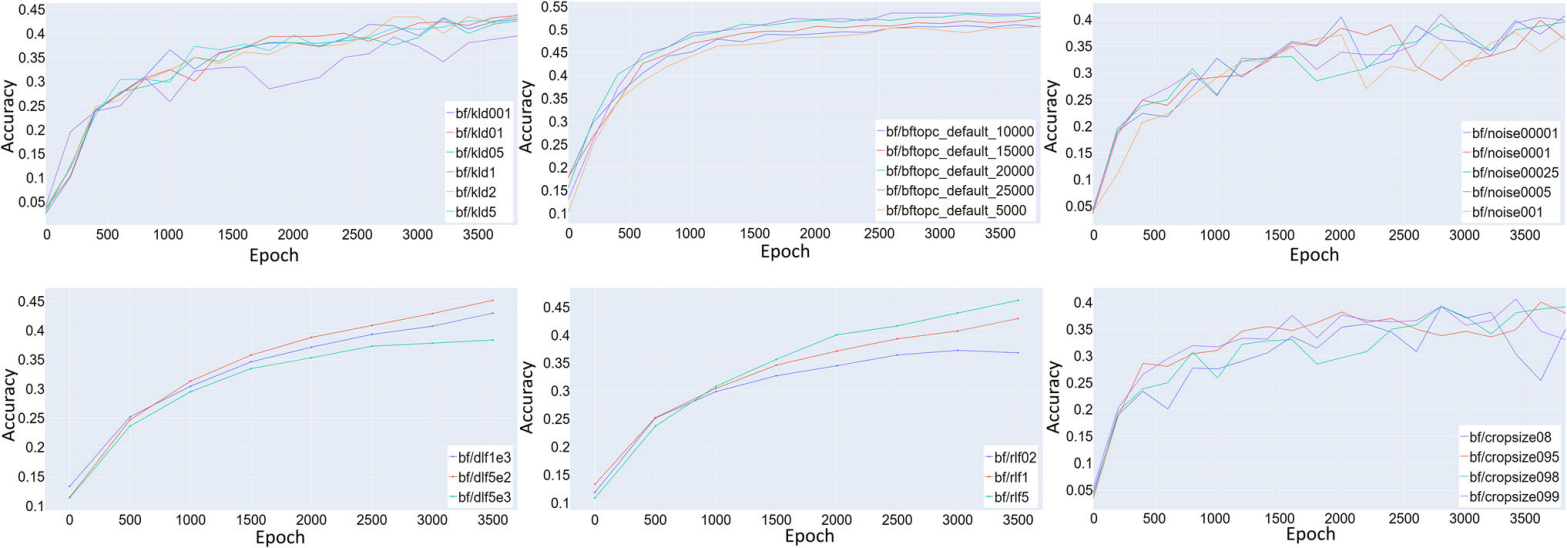
Comparison of a variety of hyperparameter choices. These choices include: KLD loss factor (top left), rlf loss factor (bottom middle), dlf loss factor (bottom left), crop size (bottom right), noise factor (top right), length of pre-train (top middle)

When we write about freezing a section of the network in this work, mathematically, we multiply the gradient update $\varDelta$ of the frozen part of the network *f* with weights $w_f$ by zero. Hence, frozen weight update refers to the rule $w_f = w_f + \varDelta w_f * 0$ instead of the normal weight update $w_f = w_f + \varDelta w_f$ The gradient is still passed through to non-frozen parts of the network, enabling them to learn. Since no standard procedure exists in the literature for applying transfer learning to a Twin-VAE which is trained with synthetic data augmentation, four different possible methods of transfer learning are proposed and compared. These methods are:

*Frozen outer layers* A popular observation in convolutional networks is that the early layers consist of universal edge processing masks and the later layers are more specialized for the task at hand [29]. In a Twin-VAE architecture, these later layers of a standard convolutional network correspond to the shared inner layers and the early convolutional layers correspond to the outer layers of the Twin-VAE. Based on this analogy, we try to train only the inner layers of the network. Everything which is not part of the shared elements of the network pictured in Fig. 4 is not trained.

*Frozen core* A common view on VAEs is that the produced embedding space should be highly sensitive in regard to the variance in the training set. Since the imaging method is not changed during normal training the VAE embedding should not encompass this variable, rather it should be highly sensitive to cell count and cell position in the images, which were the main things varied in the original training set. Since the task of cell counting remains the same and the only difference between tasks is the imaging method used, we tried to keep this shared inner representation frozen during training. Everything that is part of the shared elements of the network pictured in Fig. 4 is not trained.

*Simultaneous transfer* In the third series of tests, we were not freezing any layers at all. This has the potential problem of the initial transfer period with high losses destroying useful information in intermediate layers.

*Thawing layers.* Last, we experimented to start with frozen inner or outer layers and gradually unfreeze them during training. This is done explicitly to prevent the potential problem described in Simultaneous transfer, but to still be able to fine-tune these layers appropriately to the new task.

### Hyperparameter tuning

The original Twin-VAE needed 50 000 epochs to converge to satisfactory results, which equates to a near 100 h on an NVIDIA Tesla P-100 16 G. This made hyperparameter tuning using standard methods computationally costly.

By using transfer learning to converge significantly faster to similar or even better results, we were able to conduct more extensive hyperparameter searches. Since a full grid search over all possible hyperparameters is still not computationally feasible, instead, an iterative search was performed by tuning a single hyperparameter finding the best value and proceeding to the next hyperparameter, recapturing obscured parameter choices in later repetitions. The hyperparameters and training options tuned were:

- Training time,
- Transfer method,
- Image noise ratio,
- Image crop size,
- Relative ratio of KLD loss, regression loss and reconstruction loss
- Learning rate
- Learning rate schedule

Plots for most of these are provided in Fig. 9.

### Results

We see when tuning the hyperparameters that most of the parameters show only small improvements to the final accuracy (1–2 percent), cumulatively the performance of the network can be significantly improved (5 percent). Our experiments show that for most hyperparameters, large performance degradation can be observed if they are poorly chosen (Fig. 9).

**Fig. 10:**
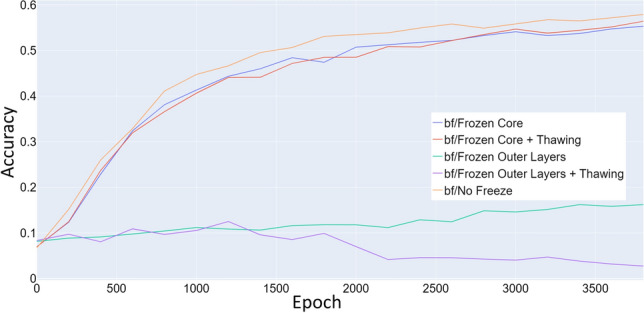
Comparison of test accuracy between different transfer learning options. The transfer was performed from the datasets Syn-PC-L-Te, Nat-PC-L-Te transferring to the datasets Syn-BF-L-Te, and Nat-BF-L-Te. In this application, the simplest option (not freezing any layers at all) performs the best, while freezing only the inner layers performs only slightly worse. Freezing the outer layers greatly impact the ability of the network to adapt to the new imaging method. Gradual thawing of the frozen layers does not have a large impact on performance

The choice of transmission method revealed which parts of the network have learned transferable information and which need to be retrained (Fig. 10). We conclude that, unlike in a typical convolutional network, the outer layers need the most retraining. If these layers remained frozen, the network could not successfully transfer its knowledge to the new imaging method. Conversely, when the inner layers remained frozen, the network achieved only 1–2 percent less performance than when everything remained unfrozen.

This could be due to the rather unique circumstance of the final task being the same, just on pictures taken with a different imaging method.

Not freezing any layers achieved the best performance overall, we attribute this to the postulated effect of the initial transfer window scrambling information not being observed.

Another interesting effect observed was that when trained for very long training times (150 000 epochs) the network did not show any signs of double descent [16] and achieved convergence after only 10 000 epochs. Compared to the non pre-trained network where convergence was achieved at the earliest after 50 000 epochs, this represents a speed up of at least 5 times.

## Twin-VAE during transfer learning

In this section, we systematically investigate the effect the unique architecture of the Twin-VAE has on the transfer process. We choose to investigate whether the decoder part of the network is needed during transfer learning, and whether the synthetic data used for training is needed during transfer learning. Through this we ask if the Twin-VAE architecture is only necessary for the pre-training part and if it can be simplified to a normal convolutional network for transfer learning.

### Decoder

**Fig. 11:**
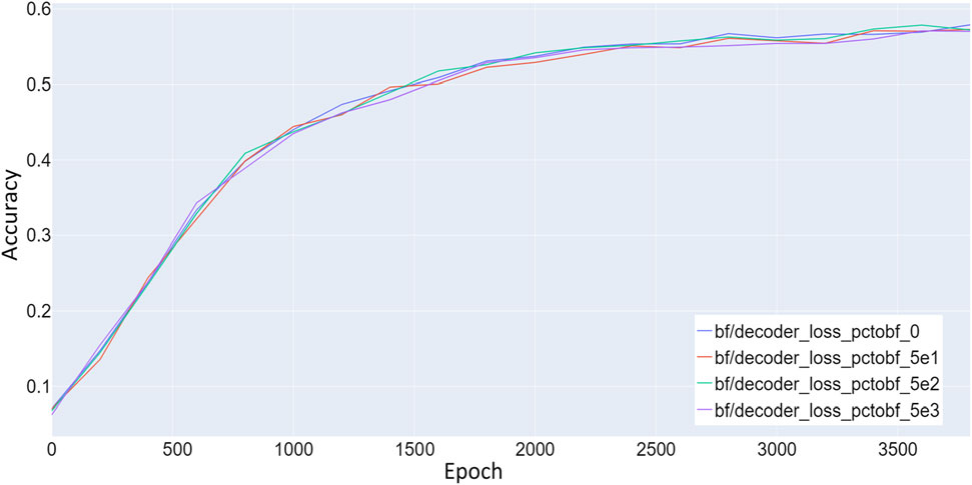
Comparison of test accuracy during transfer between different reconstruction loss factors $C_{{{\,\mathrm{\mathcal {D}_{KL}}\,}}}^{t}$. The transfer was performed from the datasets Syn-PC-L-Te, Nat-PC-L-Te transferring to the datasets Syn-BF-L-Te, and Nat-BF-L-Te. All values tried have little to no effect on the accuracy

To investigate whether the Decoder is important during transfer learning, we perform multiple transfer training runs where we successively lower the reconstruction loss factor $C_{{{\,\rm{Rec}\,}}}^{t}$. Figure 11 shows that the loss factor does not seem to have any impact on performance. The variance between runs is small enough to be within normal statistical deviations observed between runs with the same parameters and can not be clearly attributed to the different loss factors. Based on this, we conclude that the Decoder part of the network does not have a positive impact during the transfer procedure and can be set inactive to speed up transfer computing time even further.

### Synthetic data

To assess the relevance of the synthetic data during transfer learning, we vary the ratio of synthetic data to natural data. In the original paper [22] the ratio of synthetic data to natural data was maintained at 1:1 to prevent the architecture from projecting the different data types to distinct embeddings in the VAE bottleneck. A point of note is that a large part of the natural data is unlabeled, while the synthetic data are fully labeled, subsequently the synthetic data had a large contribution to the training of the regressor.

During our experiments we vary the ratio of synthetic to natural data in the range of 0.25–10:1.

**Fig. 12:**
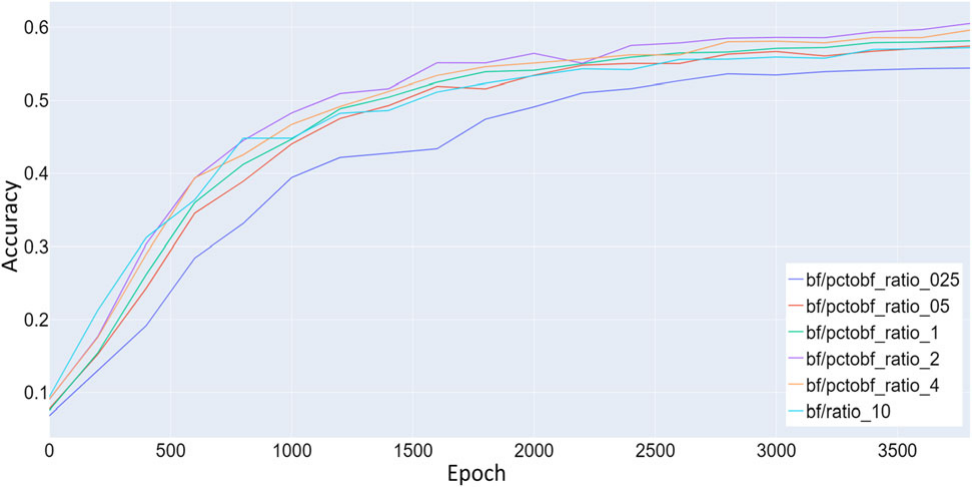
Comparison of test accuracy during transfer between different synthetic data to natural data ratios. The transfer was performed from the datasets Syn-PC-L-Te, Nat-PC-L-Te transferring to the datasets Syn-BF-L-Te, and Nat-BF-L-Te. Synthetic data ratio below 0.5:1 negatively impact the network’s performance, ratios above 5:1 also have a negative impact upon performance. The best performance was achieved with a ratio of 2:1

Figure 12 suggests that the performance of the network is negatively affected when the synthetic data ratio is especially small (<0.5) or large (>5). The optimal ratio found was 2:1. Since higher amounts of training data generally lead to better performance, there seems to be a problem generalizing from the synthetic data to the natural data.

**Fig. 13:**
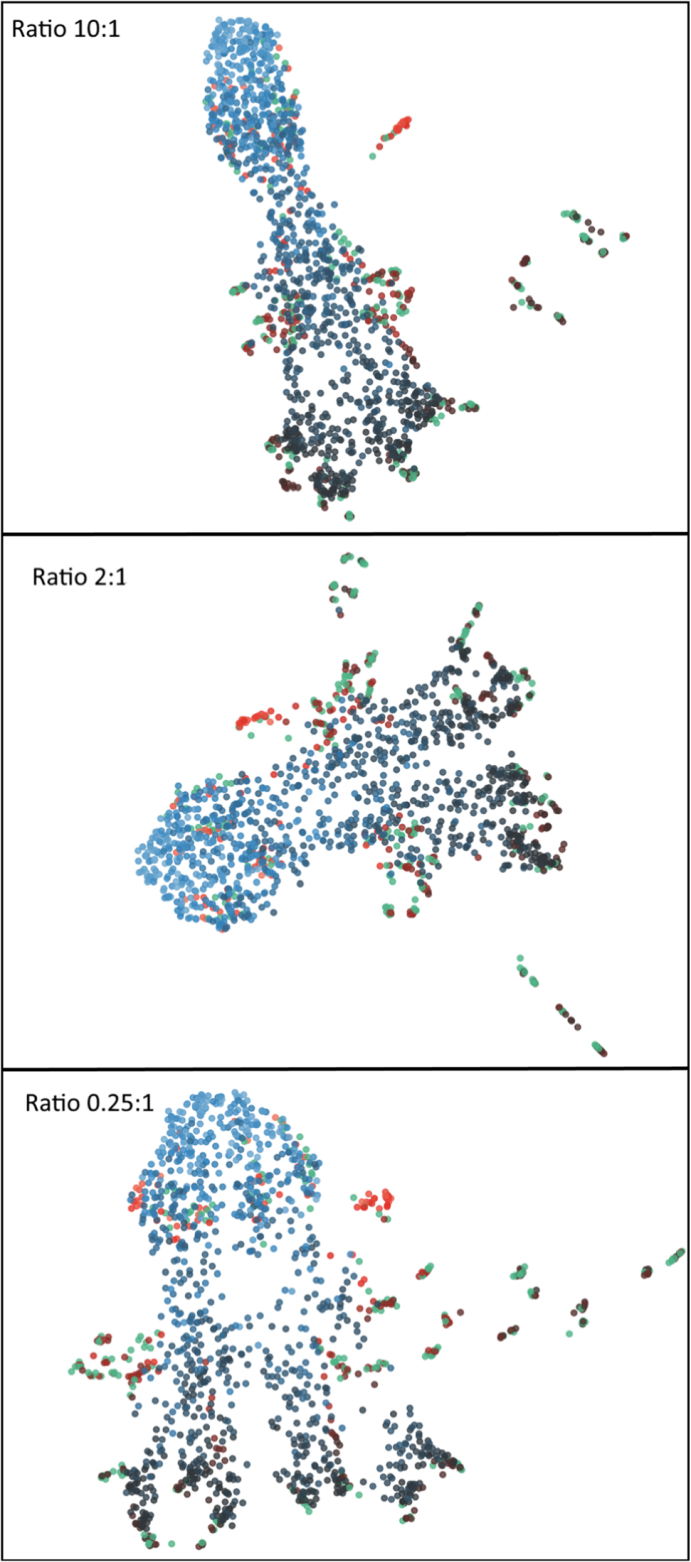
Different UMAPs of the embedding dimension of the Twin-VAE after transfer. On the dataset Syn-PC-L-Te, Nat-PC-L-Te transferring to the datasets Syn-BF-L-Te, and Nat-BF-L-Te. Blue dots represent synthetic data, red dots natural data and green dots natural unlabeled data. The color gradient of the dots represent the number of cells in the image, where lighter indicates a higher cell count. In all cases some outliers of red and green dots are found. In the lowest ratio these outliers seem to be most severe. The direction of low to high cell count is clearly recognizable in all plots. The high ratio visualization has the strongest correlation of cell count to embedding distance

We suggest that the KLD loss ${{\,\mathrm{\mathcal {D}_{KL}}\,}}$ is still able to keep the distribution of the natural and the synthetic data the same in cases where the ratio is close enough to 1:1 but for more extreme ratios the KLD loss ${{\,\mathrm{\mathcal {D}_{KL}}\,}}$ alone is insufficient. To validate this hypothesis we show 3 different UMAPs [14] in Fig. 13 that depict the distribution of natural and synthetic data in the embedding layer of the VAE.

Figure 13 shows that the VAE distribution seems to regard the number of cells in an image as a more important aspect, the higher the synthetic data ratio. However, it does not show a separation of synthetic (blue dots) and natural data (red dots), so it is not clear why the performance of the model decreases for higher synthetic data ratios. We leave this for future work.

## Results and discussion

We present the final results of all methods on the four data sets Syn-PC-L-Te, Nat-PC-L-Te, Syn-BF-L-Te, and Nat-BF-L-Te in Table 2. Our Transfer Twin-VAE consistently outperforms all other methods Twin-VAE and BiT by a clear margin on the Syn-BF-L-Te, and Nat-BF-L-Te data sets. On the Syn-PC-L-Te and Nat-PC-L-Te data sets, where more natural data are available for training, the stronger initialization by Transfer Twin-VAE does not have as strong of an impact. It has a good performance on the dataset with very little training time, but does not easily achieve the same performance as Twin-VAE on Nat-PC-L-Te. On the Syn-PC-L-Te it outperforms all other methods handily, this is most likely due to the transfer working even better on synthetic images than on natural images. Further reasons for Transfer Twin-VAE not performing better than Twin-VAE on Nat-PC-L-Te could be that the performance of the initialization on the original dataset is not as high. To remedy this, we used the Transfer Twin-VAE transferred to Nat-BF-L-Te and Syn-BF-L-Te as a starting point to transfer back to Nat-PC-L-Te and Syn-PC-L-Te, using this configuration we obtained the best results on Nat-PC-L-Te and Syn-PC-L-Te, we call this configuration the double Transfer Twin-VAE.

**Table 2:** Evaluation of all methods on the data sets Syn-PC-L-Te, Nat-PC-L-Te, Syn-BF-L-Te, and Nat-BF-L-Te.

Method	Syn MAE $\downarrow$	MRE / % $\downarrow$	Acc. / % $\uparrow$	Nat MAE $\downarrow$	MRE / % $\downarrow$	Acc. / % $\uparrow$	Training time / sec $\downarrow$
PC (phase-contrast microscopy)							
Twin-VAE (Nat only)	n/a	n/a	n/a	1.07	20.1	39.8	180000
${\texttt {Twin-VAE}}_{\texttt {max-acc}}$	**0**.**09**	0.68	68.2	0.60	5.92	57.8	400000
${\texttt {Twin-VAE}}_{\texttt {min-dev}}$	0.14	0.73	62.1	0.59	5.66	57.0	400000
BiT	n/a	n/a	n/a	2.203	n/a	26.13	9000
Transfer Twin-VAE(Nat only)	n/a	n/a	n/a	1.01	14.11	44.2	24000
Transfer Twin-VAE	0.15	0.43	85.0	0.66	6.46	53.7	40000
double Transfer Twin-VAE	0.12	**0**.**43**	**85**.**0**	**0**.**58**	**5**.**56**	**58**.**7**	71000
BF (bright-field microscopy)							
Twin-VAE (Nat only)	n/a	n/a	n/a	0.91	13.3	23.4	150000
${\texttt {Twin-VAE}}_{\texttt {max-acc}}$	0.48	4.27	60.1	0.68	7.60	53.2	310000
${\texttt {Twin-VAE}}_{\texttt {min-dev}}$	0.52	4.63	58.2	0.63	7.31	51.9	310000
BiT	n/a	n/a	n/a	1.03	n/a	43.1	5400
Transfer Twin-VAE(Nat only)	n/a	n/a	n/a	0.72	7.88	51.36	20000
Transfer Twin-VAE	**0**.**40**	**3**.**87**	**66**.**6**	**0**.**52**	**5**.**47**	**60**.**74**	31000

Numbers in bold indicate the best performance on the respective dataset and metric

For each method and data set, we report the mean absolute error (MAE), the mean relative error (MRE), and the accuracy. Ultimately, only the performance on natural data (Nat) is important, but we also report the performance on synthetic data (Syn) to provide further context. We use an upward arrow $\uparrow$ to indicate that higher is better and a downward arrow $\downarrow$ to indicate that lower is better. Training times are reported on an NVIDIA Tesla P-100 16 G GPU for all models

The most useful long-term information transfer seems to be happening from better performing microscopy methods (phase contrast) to worse performing methods (bright field). In conclusion, a magnitude shorter training times, a better starting point and some hyperparameter tuning always outperforms random weight initialization. Interestingly, the resulting transfer performance on the Syn-BF-L-Te, and Nat-BF-L-Te datasets is better than either microscopy method alone (60.74 $\leftrightarrow$ 53.20/57.80).

**Fig. 14:**
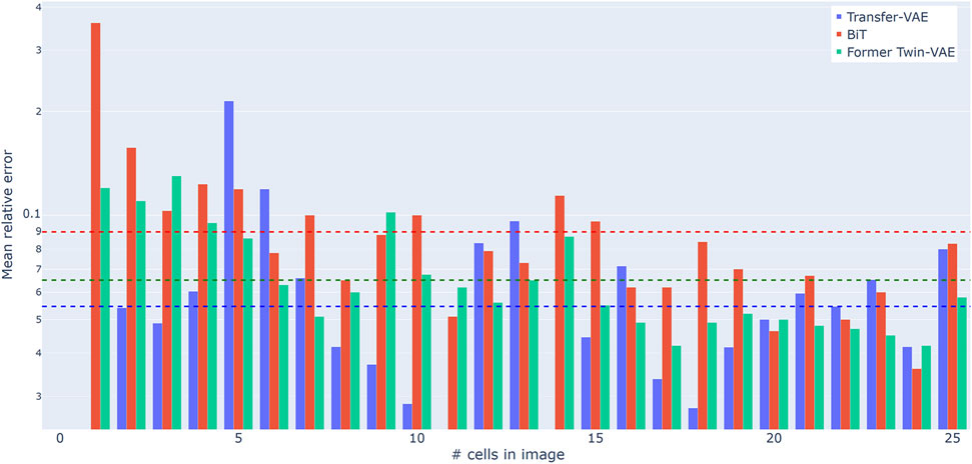
The mean relative error (MRE) for BiT, Transfer Twin-VAE, and ${\texttt {Twin-VAE}}_{\texttt {min-dev}}$ on Nat-BF-L-Te on a logarithmic scale. Horizontal bars indicate the average MRE of their respective color and method

In summary, the factors gained with our best methodology double Transfer Twin-VAE are: 19% better accuracy compared to BiT on the Nat-BF-L-Te dataset, 32% accuracy better accuracy compared to BiT on the Nat-PC-L-Te dataset. We achieved even higher accuracy gains compared to EfficientNet and Watershed. We gained about 1.1% accuracy compared to our previous Twin-VAE on the Nat-PC-L-Te dataset and about 7.5% accuracy compared to Twin-VAE on the Nat-BF-L-Te dataset (Table 2). For a more detailed performance comparison itemized by cell count see Fig. 14.

## Conclusion and outlook

In this paper, we present a significant improvement over the original Twin-VAE by using transfer learning methods to improve the accuracy and training times of the original architecture using pre-trained checkpoints of the original paper. Utilizing these shortened training times, we perform extensive hyperparameter tuning and improve the accuracy even further. Furthermore, we research which parts of the original Twin-VAE architecture are necessary for the transfer learning case. We determine that the synthetic data still plays a key role in achieving high performance and can not be removed without significant performance loss. The Decoder part of the network does not contribute to achieving higher accuracies during transfer learning and is therefore only necessary for pre-training on the original datasets.

*Limitations* The transfer procedure only performs well if the initial starting point has a high performance on the original dataset. If the starting point has low performance on the original dataset the transfer procedure might achieve lower performance than a normally trained network. In practice this is not a big problem since good starting points (in our case the ${\texttt {Twin-VAE}}_{\texttt {max-acc}}$) can be chosen easily based upon their performance on their original dataset.

The Twin-VAE architecture can use synthetic data to improve performance on natural data, currently this is limited to ratios of up to 2:1. It would be desirable if the network could abstract even further and possibly not need any labels on the natural data at all. Current methods to increase regularization (Higher ${{\,\mathrm{\mathcal {D}_{KL}}\,}}$, Dropout, Weight Decay) are not able to force the network to project the different data types onto the same embedding.

*Potential and future work* The methods described in this paper enables the automation and remote surveillance of various previously tedious and labor-intensive laboratory experiments. To use its full potential it would be interesting to implement this method as edge computing on modern microscopy hardware.

To enable edge computing computational efficiency is key, here different techniques to prune network weights or similar methods could be explored to facilitate even faster computation times.

Other possible future work includes, using active learning [3] to further refine the algorithm’s predictions and enable entirely new areas of prediction, such as the survival probability of an entire cell culture.

## Data Availability

The datasets generated during and/or analysed during the current study are available in the natural and synthetic CHO-K1 time-lapse suspension cell microscopy images (bright-field and phase-contrast) v2 repository, https://pub.uni-bielefeld.de/record/2960030.
